# Secondary Traumatic Stress and Positive Adaptation Among Social Workers: The Roles of Burnout, Compassion Satisfaction, and Perceived Social Support in a Cross-Sectional Study

**DOI:** 10.3390/healthcare14142060

**Published:** 2026-07-09

**Authors:** Fang Fu, Yumeng Zhu, Mandy Lau, Soon Noi Goh

**Affiliations:** 1 Department of Social Work, School of Social Development and Public Policy, Fudan University, Shanghai 200433, China; zhuym25@m.fudan.edu.cn; 2 Medical Social Services, Changi General Hospital, Singapore 529889, Singapore; mandy_lau@cgh.com.sg

**Keywords:** secondary traumatic stress, resilience, post-traumatic growth, Singapore, China, cross-sectional survey, occupational mental health

## Abstract

**Highlights:**

**What are the main findings?**

- Secondary traumatic stress was significantly and positively associated with post-traumatic growth.
- Burnout significantly moderated this relationship, such that the positive association between secondary traumatic stress and post-traumatic growth was weaker at higher burnout levels.
- Compassion satisfaction also significantly moderated the link between secondary traumatic stress and post-traumatic growth.
- Perceived social support showed a significant positive direct relationship with post-traumatic growth, but its moderating roles were not significant.

**What are the implications of the main findings?**

- Positive adaptation to occupational trauma should not be treated as a single construct, as resilience and post-traumatic growth may follow different relational patterns.
- Addressing burnout may be important for facilitating the growth potential associated with secondary traumatic stress in social work practice.
- Workplace interventions should strengthen supportive resources while recognizing that social support functions primarily as a direct protective asset rather than a moderator.
- Trauma-informed workforce strategies should target both risk factors and adaptive resources to support social workers’ psychological well-being.

**Abstract:**

**Objectives**: This study aimed to examine the associations of secondary traumatic stress with social workers’ positive psychological outcomes (resilience and post-traumatic growth) and to test the moderating roles of burnout, compassion satisfaction, and perceived social support. **Methods**: This study adopted a cross-sectional survey design and recruited practicing social workers from health and social care institutions in Singapore and China. A total of 673 valid questionnaires were obtained, including 354 from Singapore and 319 from China. Hierarchical regression analysis was conducted to test the direct and moderating associations among the core variables. **Results**: Secondary traumatic stress was positively associated with post-traumatic growth (β = 0.25, *p* < 0.01) with no significant association with resilience. Burnout negatively moderated the secondary traumatic stress–post-traumatic growth link (β = −0.13, *p* < 0.01) but not the secondary traumatic stress–resilience relationship. Compassion satisfaction negatively moderated the associations of secondary traumatic stress with both resilience and post-traumatic growth (β = −0.14, −0.15, *p* < 0.01). Perceived social support showed no significant moderating roles but was positively associated with both adaptation indicators (β = 0.19, 0.14, *p* < 0.01). **Conclusions**: This study challenges the conventional “risk-protection” dichotomy by revealing a nuanced stress–adaptation mechanism. Secondary traumatic stress co-occurs with post-traumatic growth (PTG) rather than baseline resilience, while compassion satisfaction exhibits a paradoxical “buffering-ceiling” interaction that attenuates the positive associations between traumatic stress and growth. Practically, organizations must move beyond generic wellness programs to implement dual-track strategies: mitigating burnout to foster social workers’ growth potential while leveraging compassion satisfaction as an early-stage shield rather than a late-stage resilience enhancer.

## 1. Introduction

### 1.1. The Occupational Burden of Secondary Traumatic Stress Among Social Workers

Social workers are inherently exposed to occupational trauma, rendering secondary traumatic stress (STS) a critical public health and occupational epidemiology concern. Secondary traumatic stress refers to the trauma-related psychological distress and physiological stress reactions arising from indirect exposure to clients’ traumatic experiences during sustained, professional interactions with survivors of violence, abuse, neglect, and systemic crises [1,2]. Globally, social workers function as critical safety nets during humanitarian and social crises; however, this role imposes a heavy psychological toll [3].

Epidemiological surveys indicate that the occupational burden of mental health challenges among social workers is severe and pervasive. Evidence suggests that the prevalence of high secondary traumatic stress among social workers can be as high as 28.8% [4]. This elevated prevalence indicates that secondary traumatic stress is not merely an individual, isolated struggle but a structural public health issue that threatens the sustainability of the social work workforce.

Traditionally, the literature on social work occupational health has been heavily skewed toward a deficit-based model, focusing primarily on negative outcomes such as professional burnout (BO), characterized by emotional exhaustion, depersonalization, and reduced personal accomplishment [5], and its co-occurrence with secondary traumatic stress.

Within the Professional Quality of Life (ProQOL) framework, secondary traumatic stress and burnout represent the negative dimensions of professional life, while compassion satisfaction (CS), the positive sense of meaning and accomplishment derived from helping others, serves as the primary internal resource [6]. Although secondary traumatic stress and burnout are highly correlated, they represent distinct epidemiological pathways: burnout arises from chronic, organizational, and administrative exhaustion, while secondary traumatic stress is an acute reaction triggered specifically by empathic engagement with traumatic material [7].

Importantly, from an epidemiological “Risk and Resilience” perspective [8,9], exposure to environmental hazards such as indirect trauma does not lead to a uniform trajectory of impairment. Instead, individuals exposed to occupational trauma exhibit highly heterogeneous adaptation pathways. While a significant subset of social workers demonstrates severe symptoms of PTSD or occupational impairment [10], others demonstrate positive psychological adaptation, manifested through resilience and post-traumatic growth (PTG). Resilience is defined as the epidemiological capacity to maintain stable, healthy psychological functioning or rapid recovery in the face of significant adversity [8,11]. In contrast, post-traumatic growth refers to subjective, positive psychological changes, such as enhanced personal strength, deeper relationships, and altered life philosophies, that emerge specifically through the cognitive struggle of processing highly challenging traumatic events [12]. Resilience and post-traumatic growth represent parallel but conceptually distinct pathways of adaptation: resilience reflects a process of “bouncing back” [13] or resisting impairment, whereas post-traumatic growth represents a transformative process of “moving forward” beyond baseline functioning.

Despite the growing body of research on social workers’ mental health, several critical gaps remain. First, existing studies predominantly focus on the direct, bivariate relationships between trauma exposure and negative outcomes, leaving the positive adaptation pathways such as resilience and post-traumatic growth under-explored within a unified epidemiological framework. Second, current literature often treats the social work population as homogeneous, failing to explore the boundary conditions that moderate the exposure–response relationships between secondary traumatic stress and adaptation. Specifically, we do not yet fully understand how internal resource depletion (burnout), internal positive resources (compassion satisfaction), and external environment resources (perceived social support, PSS) interact to condition the vulnerability or growth of social workers under traumatic stress.

To address these gaps, this study adopts a cross-sectional survey design to examine practicing social workers across two distinct East Asian social–cultural and institutional settings: Singapore and China. By investigating a total sample of 673 valid respondents, this study aims to examine the predictive effect of secondary traumatic stress on social workers’ positive psychological outcomes of resilience and post-traumatic growth and systematically test the moderating roles of burnout, compassion satisfaction, and perceived social support.

### 1.2. Theoretical Framework and Hypothesis Development

#### 1.2.1. An Integrated Theoretical Perspective

This study is theoretically grounded in the integration of the Epidemiological Risk–Resilience Framework [8], the Job Demands–Resources (JD-R) model [14], and the Conservation of Resources (COR) theory [15]. Together, these complementary theoretical perspectives provide a robust, multi-systemic framework to explain how occupational trauma exposure and resource dynamics interact to shape social workers’ positive psychological adaptation.

Within this integrated framework, secondary traumatic stress is conceptualized as an acute environmental risk exposure and a severe, resource-depleting job demand. According to the COR theory, individuals possess a finite pool of psychological and energetic resources and are motivated to acquire, preserve, and protect them [15]. The severe distress of secondary traumatic stress acts as a primary threat to these resources, potentially triggering “loss spirals” where initial resource depletion leaves social workers increasingly vulnerable to further psychological and professional impairment.

Conversely, the JD-R model posits that while job demands such as secondary traumatic stress initiate a health-impairment process, the presence of job and personal resources initiates a parallel motivational process that fosters positive adaptation and work engagement [14]. By combining these perspectives with the Epidemiological Risk–Resilience Framework, we propose that positive adaptation under occupational trauma is neither uniform nor static; rather, the exposure–response relationship between secondary traumatic stress and adaptation outcomes is highly contingent upon critical internal and external boundary conditions.

#### 1.2.2. Secondary Traumatic Stress and Dual Pathways of Positive Adaptation

Positive psychological adaptation under trauma is increasingly recognized as a non-unitary construct consisting of distinct pathways [16]. In this study, we distinguish between resilience—the homeostatic capacity to maintain psychological stability and functional integrity despite adversity [8]—and post-traumatic growth—the transformative positive psychological change resulting from the cognitive struggle with highly challenging life circumstances [17].

Under the COR theory, severe and chronic secondary traumatic stress directly drains a social worker’s energetic resources, compromising their ability to maintain psychological equilibrium and leading to a decline in baseline resilience. However, under the salutogenic framework of trauma psychology, this same distress can paradoxically serve as a cognitive catalyst [18]. When secondary trauma exposure is sufficiently intense, it delivers a profound cognitive shock that shatters a practitioner’s core assumptions about world safety and fairness [19]. This cognitive disruption, while painful, forces active cognitive processing and meaning reconstruction, which can lead to positive, transformative changes in personal and professional domains. Thus, we propose that secondary traumatic stress relates differentially to stable recovery versus transformative growth:

**Hypothesis** **1.** *Secondary traumatic stress is differentially associated with social workers’ positive psychological adaptation outcomes*.

**H1a.**  *Secondary traumatic stress is negatively associated with resilience (reflecting the resource-depletion distress pathway)*.

**H1b.**  *Secondary traumatic stress is positively associated with post-traumatic growth (reflecting the struggle-induced transformative pathway)*.

#### 1.2.3. Burnout as a Resource-Depletion Vulnerability Factor

As a core negative dimension of professional quality of life, burnout represents a state of chronic, systemic psychological and physical resource depletion [6]. Within the COR framework, individuals experiencing high burnout operate with severely compromised “resource reservoirs” [15]. When social workers are emotionally exhausted and cognitively depleted by burnout, they lack the necessary “cognitive surplus” or psychological energy required to process and integrate traumatic client narratives.

Consequently, rather than utilizing secondary distress to fuel reflection and cognitive restructuring, burnt-out social workers are trapped in resource loss spirals, rendering them highly vulnerable to the destabilizing effects of secondary traumatic stress and suppressing their capacity for meaning making [20]. Burnout is therefore expected to act as a significant negative boundary condition that worsens distress and paralyzes growth.

**Hypothesis** **2.** *Burnout moderates the relationships between secondary traumatic stress and positive adaptation, acting as an internal resource-depletion vulnerability*.

**H2a.**  *Burnout amplifies the negative association between secondary traumatic stress and resilience*.

**H2b.**  *Burnout dampens the positive association between secondary traumatic stress and post-traumatic growth*.

#### 1.2.4. Compassion Satisfaction as an Internal Protective Resource

In contrast to burnout, compassion satisfaction represents a powerful internal personal resource, reflecting the emotional rewards, professional self-efficacy, and existential meaning that social workers derive from helping others [6]. According to the JD-R model, such personal resources can buffer the negative impact of high job demands on psychological strain [14]. High compassion satisfaction provides a positive emotional reserve and bolsters professional self-efficacy, safeguarding social workers’ baseline stability (resilience) when facing secondary trauma.

However, the moderating role of compassion satisfaction on growth-related pathways may be more complex. Highly satisfied individuals already possess stable, highly integrated cognitive assumptions about their professional efficacy and world order. Consequently, high compassion satisfaction may shield social workers so effectively that secondary trauma exposure fails to deliver the “cognitive shock” necessary to shatter their core assumptions, thereby reducing the necessity for active cognitive rebuilding and weakening the slope of the trauma-to-growth pathway [19,21]. We therefore hypothesize:

**Hypothesis** **3.** *Compassion satisfaction moderates the relationships between secondary traumatic stress and positive adaptation, acting as an internal psychological buffer*.

**H3a.**  *Compassion satisfaction weakens the negative association between secondary traumatic stress and resilience*.

**H3b.**  *Compassion satisfaction strengthens the positive association between secondary traumatic stress and post-traumatic growth*.

#### 1.2.5. Perceived Social Support as an External Ecological Resource

While positive adaptation is often measured at the individual level, socio-ecological frameworks increasingly position resilience and growth as ecologically embedded processes shaped by interpersonal and organizational environments [9]. Within the Epidemiological Risk–Resilience Framework, perceived social support represents a primary external environmental asset [8].

For social workers exposed to chronic occupational trauma, social support—spanning reflective peer supervision, administrative appreciation, and informal collegial validation—provides the essential relational “scaffolding” to navigate distress [22]. When social workers perceive strong social support, they do not have to process secondary traumatic narratives in isolation; the supportive environment facilitates safe cognitive processing, reinforces professional belonging, and buffers against the destabilizing effects of secondary traumatic stress while actively promoting growth-oriented meaning-making. We therefore hypothesize:

**Hypothesis** **4.** *Perceived social support moderates the relationships between secondary traumatic stress and positive adaptation, acting as an external environmental scaffolding*.

**H4a.**  *Perceived social support weakens the negative association between secondary traumatic stress and resilience*.

**H4b.**  *Perceived social support strengthens the positive association between secondary traumatic stress and post-traumatic growth*.

**Figure 1:**
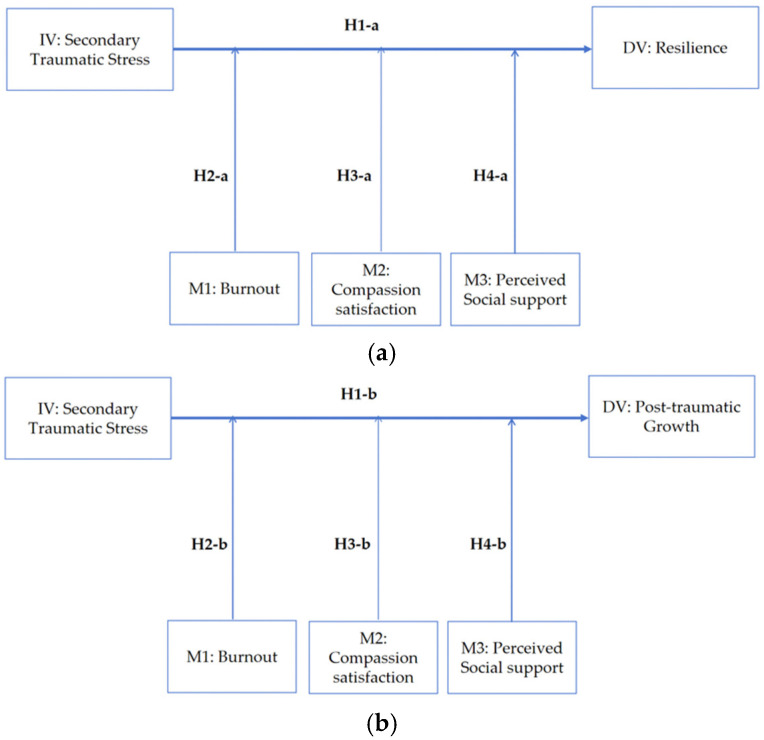
(**a**). Hypothesized conceptual model between secondary traumatic stress and resilience. (**b**). Hypothesized conceptual model between secondary traumatic stress and post-traumatic growth.

The hypothesized conceptual model is presented in Figure 1.

## 2. Materials and Methods

### 2.1. Study Design, Setting, and Participants

This study employed a cross-sectional, observational survey design to examine social workers’ professional experience and psychological adaptations. The reporting of this study adhered strictly to the Strengthening the Reporting of Observational Studies in Epidemiology (STROBE) guidelines. A complete STROBE checklist is available in Supplementary Materials.

The data analyzed in the present study were drawn from a larger collaborative research project spanning seven jurisdictions during the COVID-19 pandemic, which investigated social workers’ work role transitions, virus exposure, cognitive fears, and psychological outcomes. The initial broad findings of this collaborative project were published in a dedicated Special Issue of *Social Work in Health Care* [11,23,24]. Building upon this collaborative foundation, the present study represents a targeted, deeper collaborative analysis specifically comparing social workers from Singapore and China. The primary aim is to systematically model the conditional pathways (i.e., the “trauma–exhaustion–growth” continuum) through which social workers achieve positive psychological adaptation under varying degrees of secondary traumatic stress exposure.

Participants were recruited via online snowball sampling. Given the logistical barriers, lock-downs, and administrative constraints during the pandemic, online non-probability sampling represented a highly pragmatic and efficient approach to recruit frontline practitioners across geographically dispersed regions. Data collection was facilitated through professional web-based survey platforms from August to September 2020 in Singapore, and in January 2021 in China.

For the Singapore cohort, invitation emails containing the survey link were distributed in partnership with the Singapore Association of Social Workers (SASW) and the heads of social work departments across major healthcare and community care institutions. For the Chinese cohort, recruitment invitations were disseminated nationwide to the chairpersons of regional social work committees via a dedicated professional WeChat network. These chairpersons subsequently forwarded the invitations to localized WeChat groups comprising social work department directors, who then invited frontline social workers meeting the study’s inclusion criteria to participate. All participants provided informed consent electronically before accessing the anonymous questionnaire. The final analytical sample comprised 673 practicing social workers, including 354 from Singapore and 319 from China.

Institutional Review Board (IRB) approvals were obtained from the SingHealth Central Institutional Review Board (Singapore) and the School of Social Development and Public Policy Institutional Review Board in Fudan University (China) prior to study initiation.

### 2.2. Bias and Study Size

To minimize potential methodological biases, several proactive steps were implemented. First, to mitigate social desirability and evaluation apprehension biases, all questionnaires were administered completely anonymously online. Second, established, psychometrically validated instruments were utilized to ensure measurement consistency. Potential limitations inherent to cross-sectional survey designs, such as selection bias, recall bias, and common method variance, were statistically monitored and are addressed in the discussion.

Regarding study size adequacy, the final sample of 673 was evaluated against established psychometric and multivariate analysis recommendations. For a model with six primary variables and 48 total item indicators, this sample size far exceeds the conservative rule-of-thumb ratio of 5 to 10 participants per item, yielding a required range of 240 to 480 cases. This indicates that the study is robustly powered to perform complex multivariate moderate regression analyses.

### 2.3. Variables and Measurements

#### 2.3.1. Professional Quality of Life (ProQOL)

Social workers’ secondary traumatic stress, burnout, and compassion satisfaction were assessed using the Professional Quality of Life (ProQOL, Version 5) [6]. The ProQOL is a 30-item self-report scale comprising three 10-item subscales.

Secondary traumatic stress assesses the negative psychological and physiological impact of indirect exposure to clients’ traumatic experiences (e.g., “I think that I might have been affected by the traumatic stress of those I help.”). In this study, Cronbach’s α for the secondary traumatic stress subscale was 0.76.

Burnout measures feelings of hopelessness, cognitive exhaustion, and occupational strain (e.g., “I feel trapped by my job as a helper”). Cronbach’s α for the burnout subscale was 0.81.

Compassion satisfaction measures the positive aspects of trauma work, specifically the professional fulfillment and sense of efficacy derived from helping others (e.g., “I get satisfaction from being able to help people”). Cronbach’s α for the compassion satisfaction subscale was 0.92. All items were rated on a 5-point Likert scale from 1 (Never) to 5 (Very Often). In strict accordance with the official ProQOL-5 scoring manual, each subscale was analyzed as an independent construct, and no aggregate or cumulative “overall ProQOL” score was calculated.

#### 2.3.2. Resilience

Resilience was assessed using the 2-item Connor–Davidson Resilience Scale (CD-RISC-2) [25], which captures core features of coping capacity (items: “Able to adapt to change” and “Tend to bounce back after illness or hardship”). Items were scored on a 5-point Likert scale from 0 (Not true at all) to 4 (True nearly all of the time). The reliability coefficient in this study was 0.76.

#### 2.3.3. Post-Traumatic Growth

Post-traumatic growth was assessed using the 10-item abbreviated Post-Traumatic Growth Inventory (PTGI-SF) [26]. The scale measures five distinct growth dimensions: relating to others, new possibilities, personal strength, spiritual change, and appreciation of life. Items were rated on a 6-point Likert scale from 0 (“I did not experience this change as a result of my crisis”) to 5 (“I experienced this change to a very great degree as a result of my crisis”). Cronbach’s α in this study was 0.94.

#### 2.3.4. Perceived Social Support

Perceived Social Support was measured using the concise 6-item questionnaire on Social Support (F-SozU K-6) [27]. Items were scored on a 5-point Likert scale ranging from 1 (Not true at all) to 5 (Very true), with higher scores representing stronger perceived interpersonal and social scaffolding. Cronbach’s α in the current study was 0.88.

### 2.4. Language Equivalence and Translation

The survey was administered in English for the Singapore sample and in Simplified Chinese for the China sample. To ensure semantic, conceptual, and linguistic equivalence across both regions, the Chinese version was developed using a rigorous standard translation and back-translation procedure conducted by bilingual social work scholars.

### 2.5. Ethical Licensing and Copyright Declarations

ProQOL-5 License: The authors confirm that the ProQOL-5 [6] was used under the free academic and non-commercial license provided by the copyright owner (B. Hudnall Stamm, © 2010; www.proqol.org). No modifications were made to the items or scoring algorithms.

CD-RISC-2 License: The 2-item Connor–Davidson Resilience Scale (CD-RISC-2) [25] was utilized with permission and appropriate citation of the copyright holders (© Jonathan R. T. Davidson and Katherine M. Connor, 2003).

### 2.6. Statistical Methods

Data management and analyses were performed using SPSS Version 25.0. Missing values for demographic variables (marital status and age) represented less than 10% of the total dataset and were managed using mode imputation. Categorical control variables (marital status and age group) were dummy coded prior to analysis.

In accordance with standard statistical practices for testing moderation [28,29], the independent variables (secondary traumatic stress) and all three moderating variables (burnout, compassion satisfaction, and perceived social support) were mean-centered prior to model estimation to reduce potential multicollinearity and improve the interpretability of the regression coefficients. The dependent variables (resilience and post-traumatic growth) remained uncentered.

Preliminary analyses included descriptive statistics for participants’ demographic characteristics, Pearson correlation analyses, evaluation of the measurement model, and multicollinearity diagnostics.

To test the moderating effects, hierarchical regression analyses were conducted. Resilience and post-traumatic growth were entered as dependent variables in separate models to examine whether secondary traumatic stress was associated with these outcomes and whether compassion satisfaction, burnout, and perceived social support moderated these relationships.

## 3. Results

### 3.1. Demographic Description

In the China sample (n = 319), females accounted for 71.2% (n = 227), and males 28.8% (n = 92). In the Singapore sample (n = 354), females comprised 78.0% (n = 276), and males 22.0% (n = 78). Regarding marital status, married participants were the largest group in both China (62.4%, n = 199) and Singapore (53.7%, n = 190), followed by single (unmarried) participants (China: 33.5%, n = 107; Singapore: 43.2%, n = 153). In terms of age, the majority were aged 21–40 years in both China (77.4%, n = 247) and Singapore (67.8%, n = 240) samples, followed by those aged 41–60 years (China: 21.9%, n = 70; Singapore: 30.8%, n = 109) (Table 1).

### 3.2. Descriptive Statistics, Normality, and Correlations

The means, standard deviations, skewness, and kurtosis values of the study variables are reported in Table 2. All items showed acceptable univariate normality, with absolute skewness values below 3 and absolute kurtosis values below 8, suggesting that the data were suitable for subsequent parametric analyses.

Pearson correlation analysis was conducted. Results revealed significant correlations: STS showed a positive correlation with PTG (r = 0.18, *p* < 0.01) and BO (r = 0.45, *p* < 0.01) while demonstrating negative correlations with resilience (r = −0.18, *p* < 0.01) and PSS (r = −0.17, *p* < 0.01). PTG was positively correlated with resilience (r = 0.14, *p* < 0.01), CS (r = 0.21, *p* < 0.01), and PSS (r = 0.18, *p* < 0.01), but negatively correlated with BO (r = −0.10, *p* < 0.05). Resilience exhibited positive correlations with compassion satisfaction (r = 0.46, *p* < 0.01) and perceived social support (r = 0.38, *p* < 0.01), yet a negative correlation with BO (r = −0.41, *p* < 0.01) (Table 3).

### 3.3. Measurement Model, Common Method Bias, and Multicollinearity Diagnostics

Firstly, to examine the distinctiveness of the focal constructs, a confirmatory factor analysis (CFA) was conducted for the six study variables: secondary traumatic stress, burnout, compassion satisfaction, perceived social support, resilience, and post-traumatic growth. The hypothesized six-factor measurement model exhibited acceptable absolute fit to the data (χ^2^/df = 4.66, SRMR = 0.09, RMSEA = 0.07), though increment fit indices fell below the conventional 0.90 threshold (CFI = 0.80, TLI = 0.80). This pattern is common in complex measurement models with a large number of factors and indicators, where incremental indices are prone to downward bias [30]. To explore whether the lower CFI and TLI values were attributable to model complexity rather than misspecification, we conducted an auxiliary CFA using item parceling in random algorithm [31]. The parcel-based model yielded improved incremental fit indices (CFI = 0.96, TLI = 0.95), corroborating that the original model’s absolute fit indices provide adequate evidence of construct distinctiveness. Because the primary analyses in this study utilize manifest variable moderated regressions, the CFA functions as an auxiliary diagnostic for construct distinctiveness. The robust empirical distinctiveness of the variables is primarily established through the heterotrait–monotrait (HTMT) ratios and multicollinearity diagnostics reported below.

To assess the potential presence of common method bias, Harman’s single-factor test was performed. The first unrotated factor accounted for 24.73% of the total variance, which was below the conventional threshold of 40%, suggesting that common method bias was unlikely to pose a serious threat to the interpretation of the findings.

Discriminant validity was assessed using the heterotrait–monotrait (HTMT) ratio of correlations. All HTMT values were below the recommended threshold of 0.90, supporting the discriminant validity of the constructs in this study.

Multicollinearity diagnostics showed that the variance inflation factor (VIF) values of all variables were less than 5, indicating no serious multicollinearity in the models, and that the regression coefficients were stable and reliable (Table 4).

### 3.4. The Moderating Effects of Burnout, Compassion Satisfaction, and Perceived Social Support Between Secondary Traumatic Stress and Resilience

Regarding direct associations, compassion satisfaction (β = 0.32, *p* < 0.001) and social support (β = 0.19, *p* < 0.001) exhibited significant positive associations with resilience, whereas the direct association for secondary traumatic stress was not statistically significant (Table 5).

Further moderation analysis revealed that compassion satisfaction played a significant negative moderating role in the relationship between secondary traumatic stress and resilience (β = −0.14, *p* < 0.01). The interaction terms involving burnout and perceived social support were not statistically significant. These results indicate that the association between secondary traumatic stress and resilience varied according to the level of compassion satisfaction.

**Figure 2:**
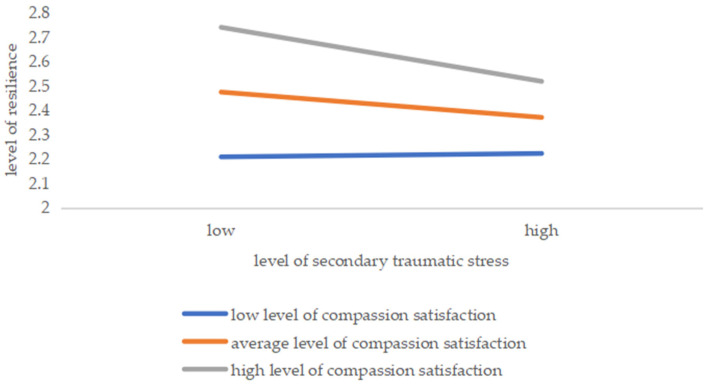
Simple Slope Plot of Compassion Satisfaction Moderating the Relationship between Secondary Traumatic Stress and Resilience.

Simple slope analyses were further conducted to probe the significant interaction. As shown in Figure 2, the association between secondary traumatic stress and resilience differed at low (−1 SD) and high (+1 SD) levels of compassion satisfaction. Specifically, when compassion satisfaction was low, the slope representing the association between secondary traumatic stress and resilience was positive but not significant (b = 0.007, SE = 0.037, *p* = 0.852). When compassion satisfaction was high, this slope was negative and significant (b = −0.110, SE = 0.036, *p* = 0.002). This pattern indicates that the association between secondary traumatic stress and resilience differed across levels of compassion satisfaction, with a significant negative association observed only at high levels of compassion satisfaction. That is, the negative association between secondary traumatic stress and resilience was significant only among individuals with high levels of compassion satisfaction.

### 3.5. The Moderating Effects of Burnout, Compassion Satisfaction, and Perceived Social Support Between Secondary Traumatic Stress and Post-Traumatic Growth

A second hierarchical regression analysis revealed that in Step 1, gender, nationality, age, and marital status were not significantly associated with post-traumatic growth among social workers. After controlling for these demographic variables, Step 2 introduced the main effect model, demonstrating that both secondary traumatic stress (β = 0.25, *p* < 0.001) and social support (β = 0.14, *p* < 0.01) emerged as significant positive correlates of post-traumatic growth. Step 3, which examined moderating effects, further indicated that both the interaction between secondary traumatic stress and compassion satisfaction (β = −0.15, *p* < 0.01) and the interaction between secondary traumatic stress and burnout (β = −0.13, *p* < 0.01) were significant (Table 6).

**Figure 3:**
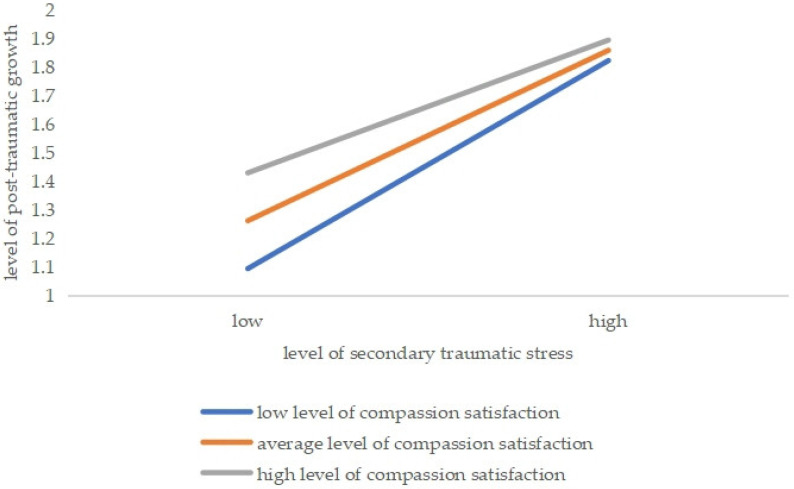
Simple Slope Plot of Compassion Satisfaction Moderating the Relationship between Secondary Traumatic Stress and Post-Traumatic Growth.

To facilitate interpretation, simple slope analyses were conducted for the significant interactions. As shown in Figure 3, the positive association between secondary traumatic stress and post-traumatic growth was weaker at high levels of compassion satisfaction (+1 SD) than at low levels of compassion satisfaction (−1 SD). Specifically, the slope for low compassion satisfaction was b = 0.365, SE = 0.071, *p* < 0.001, whereas the slope for high compassion satisfaction was b = 0.233, SE = 0.068, *p* = 0.001.

**Figure 4:**
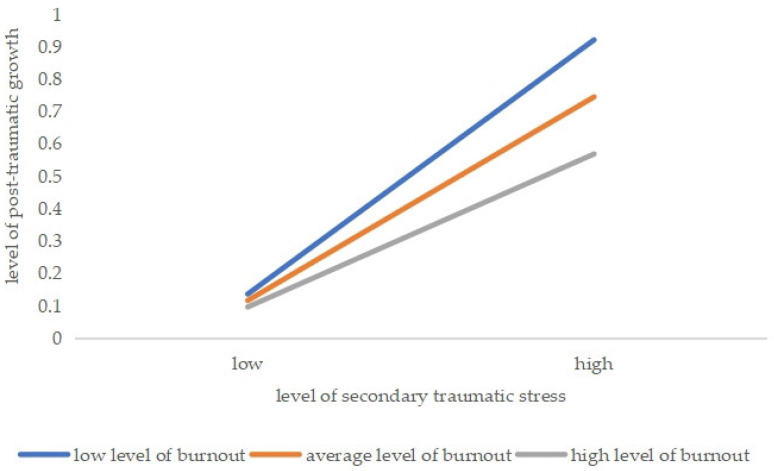
Simple Slope Plot of Burnout Moderating the Association between Secondary Traumatic Stress and Post-Traumatic Growth.

Similarly, as shown in Figure 4, the positive association between secondary traumatic stress and post-traumatic growth was stronger at low levels of burnout (−1 SD) than at high levels of burnout (+1 SD). The simple slope for low burnout was b = 0.392, SE = 0.079, *p* < 0.001, whereas the slope for high burnout was b = 0.236, SE = 0.067, *p* < 0.001.

### 3.6. Comparison of the Moderating Effects of Psychological Resilience and PTG

The two dependent variables showed different patterns of association with secondary traumatic stress and the proposed moderators (Table 7). For resilience, compassion satisfaction, and perceived social support showed significant positive associations, whereas the association with secondary traumatic stress was not statistically significant after covariates were controlled. Among the three moderators, only compassion satisfaction significantly moderated the relationship between secondary traumatic stress and resilience. For PTG, in contrast, secondary traumatic stress and perceived social support both showed significant positive associations. In addition, both compassion satisfaction and burnout significantly moderated the association between secondary traumatic stress and post-traumatic growth. Overall, the results indicated different patterns across the resilience and post-traumatic growth models with respect to the significant direct associations and interaction patterns.

## 4. Discussion

This study advances the literature on occupational trauma by mapping the complex relationships of positive psychological adaptation among social workers operating in high-trauma exposure environments. Specifically, we examined the intricate dynamics between secondary traumatic stress, burnout, compassion satisfaction, perceived social support, and positive adaptation.

Crucially, our findings resolve a persistent conceptual debate by demonstrating that resilience and post-traumatic growth represent two parallel, conceptually distinct forms of positive adaptation, rather than interchangeable facets of a singular recovery process. This is empirically evidenced by their divergent relationships with secondary traumatic stress: while secondary traumatic stress was positively associated with post-traumatic growth, its relationship with resilience became statistically non-significant once confounding covariates were controlled. This divergence indicates that resilience (the capacity to maintain stability) and post-traumatic growth (the transformative process of thriving post-adversity) are linked to fundamentally different psychological processes and resource structures [16,21].

### 4.1. Secondary Traumatic Stress and Post-Traumatic Growth: The Paradox of Growth Through Distress

The positive association between secondary traumatic stress and post-traumatic growth indicates that distress and growth dynamically coexist among social workers, aligning with the salutogenic framework of trauma psychology [18]. Rather than implying that secondary traumatic stress is universally beneficial, this relationship is best explained by a curvilinear (inverted-U) model of stress adaptation. In this sample, secondary trauma exposure likely remained within a “tolerable range,” which is theoretically associated with a level of cognitive challenge sufficient to disrupt core assumptions [19] and foster active meaning making [32], without being linked to complete psychological collapse. This non-linear mechanism is well-supported by meta-analytic evidence showing that moderate distress is associated with enhanced cognitive processing and subsequent post-traumatic growth, whereas extremely low or high distress levels are related to lower levels of the growth process [33,34].

However, post-traumatic growth should not be interpreted as a simple, uniformly positive outcome or an indicator of objective clinical recovery. Instead, it reflects self-perceived psychological changes rather than objective functional or professional improvements [21]. This distinction is central to the “Janus-face” model of post-traumatic growth [35], which posits that self-reported growth often coexists with ongoing psychological distress, serving as a constructive cognitive coping strategy rather than a sign of complete professional adaptation. Thus, secondary traumatic stress possesses a dual character, being associated with severe occupational hazards while also serving as a latent, subjective facilitator of professional cognitive transformation [18].

### 4.2. Resilience as a Resource-Related Outcome

In contrast to post-traumatic growth, resilience was not significantly associated with secondary traumatic stress in our adjusted model. Instead, compassion satisfaction and perceived social support emerged as the primary factors positively associated with resilience. Grounded in Hobfoll’s Conservation of Resources (COR) theory, this pattern suggests that resilience is not merely a reactive shield against trauma exposure but a resource-driven outcome. Rather than being solely associated with the severity of the stressor (secondary traumatic stress) itself, a social worker’s resilience appears to be more closely linked to a “resource caravan” of personal fulfillment (compassion satisfaction) and interpersonal assets (perceived social support).

The findings challenge the individualistic view of resilience, aligning with socio-ecological frameworks that position resilience as a systemic construct embedded within relational and organizational networks [36]. Consequently, supporting trauma-exposed practitioners requires organizations to shift from focusing solely on minimizing trauma exposure to actively cultivating resource-rich environments [37]. Systemic interventions, such as structured clinical supervision, peer-support networks, and organizational recognition, are essential for replenishing social workers’ relational resources and sustaining their professional resilience over time [38].

### 4.3. The Moderating Roles of Burnout, Compassion Satisfaction, and Perceived Social Support

Our moderation analyses offer a more granular look at the boundaries of psychological adaptation. First, burnout significantly moderated the positive “STS-PTG” association, such that the relationship was weaker at higher levels of burnout. Under the COR theory, burnout represents a state of severe, chronic resource depletion [15]. When social workers are emotionally exhausted and cognitively depleted by burnout, they lack the “cognitive surplus” or psychological energy required for deliberate rumination and cognitive restructuring, both of which are indispensable cognitive processes for facilitating post-traumatic growth in the context of secondary distress [16]. Therefore, mitigating burnout is not only crucial for preventing impairment but is also a functional prerequisite for preserving the cognitive capacity needed for adaptive growth.

Second, compassion satisfaction displayed a complex, non-linear moderating role. Specifically, the positive association between secondary traumatic stress and post-traumatic growth was weaker under conditions of higher compassion satisfaction. This suggests that compassion satisfaction does not function as a simple, uniform buffer. From a cognitive perspective, exceptionally high compassion satisfaction may be associated with highly stable core beliefs, meaning that secondary trauma is less likely to challenge assumptions or relate to the cognitive restructuring characteristics of the post-traumatic growth process [19]. Regarding the resilience pathway, although the interaction was statistically significant, this finding warrants cautious interpretation given that secondary traumatic stress did not exhibit a significant direct association with resilience in the adjusted model. This pattern indicates that the relationship between secondary traumatic stress and resilience is highly conditional—manifesting only at high levels of compassion satisfaction—rather than representing a uniform, direct link across all practitioners. This finding underscores that growth and stable adaptation (resilience) are associated with distinct psychological processes, with compassion satisfaction showing divergent roles depending on whether the cognitive state of interest is meaning reconstruction (growth) or homeostasis (resilience).

Finally, while perceived social support was strongly and directly associated with psychological adaptations, it did not moderate the relationships between secondary traumatic stress and resilience, or between STS and PTG. This distinction aligns with Cohen and Wills’ (1985) classic social support framework, which differentiates between the “direct-effect hypothesis” and the “buffering hypothesis” [39]. In this sample, perceived social support functioned as a robust “background resource,” associated with higher overall baseline resilience regardless of trauma levels, but it did not moderate the relationship between secondary trauma and positive adaptation [40]. Thus, while general social and organizational support is indispensable for basic well-being, specialized, trauma-informed clinical interventions remain necessary to support the cognitive transition from distress to growth.

### 4.4. Null Findings and Inconsistent Moderation: Theoretical and Methodological Reflections

Several hypothesized moderation relationships were not supported by our empirical data, warranting careful theoretical and methodological reflection.

First, the non-significant moderating effect of burnout on the STS–resilience relationship (H2a) suggests that burnout may operate not as a conditional vulnerability boundary but as a pervasive, resource-depleting condition consistently associated with lower resilience across all levels of trauma exposure. Under the COR theory [15], chronic burnout represents a global state of depletion that limits overall psychological functioning rather than a specific variable altering the strength of the association between secondary traumatic stress and psychological resilience. Consequently, the co-occurrence of high burnout and low resilience remains stable, independent of trauma severity.

Second, the lack of moderating associations for perceived social support (H4a, H4b) aligns with the direct-effects hypothesis rather than the buffering hypothesis [41]. Methodologically, this may stem from our measurement instrument—the F-SozU K-6 scale—which captures generalized support from family and friends, failing to differentiate informal social networks from formal, workplace-specific professional support (e.g., clinical supervision). While informal support is strongly associated with baseline well-being, it often lacks the specialized clinical depth necessary to help practitioners cognitively reframe occupational trauma.

Furthermore, generalized social support may facilitate emotional venting rather than deliberate rumination—the conscious cognitive processing required to link distress with post-traumatic growth. Consequently, while social support remains a vital positive correlate of overall adaptation, organizations cannot rely solely on general networks to buffer secondary traumatic stress. Specialized, trauma-informed clinical frameworks are necessary to support structured cognitive adaptation and growth in this population.

### 4.5. Theoretical Implications

This study makes three primary theoretical contributions to the literature on occupational trauma and positive adaptation in the helping professions.

First, it advances a multi-dimensional, non-unitary conceptualization of positive adaptation. By demonstrating that resilience and post-traumatic growth represent parallel but distinct processes, this study challenges the assumption that positive post-trauma adaptation is a homogeneous construct [16]. Our finding that secondary traumatic stress was positively associated with post-traumatic growth but had no significant relationship with resilience in the adjusted model suggests that secondary trauma exposure can be linked to transformative meaning making without necessarily being associated with a practitioner’s homeostatic emotional stability [21]. Researchers must therefore distinguish between stable recovery (resilience) and transformative growth when modeling occupational trauma adaptation.

Second, this study theoretically synthesizes the Professional Quality of Life framework with the Conservation of Resources theory. While the ProQOL framework maps occupational strains (secondary traumatic stress, burnout) and rewards (compassion satisfaction), COR theory provides the explanatory framework for their interactive dynamics. Our finding that burnout significantly moderates the relationship between STS and PTG—such that the association is weaker under high levels of burnout—empirically illustrates “resource depletion” in action: when emotional and cognitive resources are limited (as characterized by high burnout), the cognitive bandwidth associated with processing distress in relation to growth is severely compromised. Conversely, compassion satisfaction may function as an internal psychological resource linked to a stable worldview, which is associated with a different pattern of cognitive restructuring [42].

Third, this study bridges organizational behavior and trauma psychology by integrating these findings into the Job Demands–Resources (JD-R) model. Within this unified framework, secondary traumatic stress and burnout function as job demands associated with the health-impairment process, while perceived social support and compassion satisfaction act as job resources linked to the motivational and adaptive process. By embedding trauma-related variables of interest into the JD-R model, we provide a systemic, structurally grounded framework through which organizations can support and facilitate sustainable professional functioning under chronic exposure to suffering.

### 4.6. Practical Implications

Translating these findings into systemic action suggests a coordinated, multi-level intervention framework that spans organizational, supervisory, and individual levels.

At the organizational level, agencies may benefit from shifting from a passive posture of simply minimizing trauma exposure to an active, structural strategy of cultivating robust “resource caravans” [15]. As our findings demonstrate that burnout negatively moderates the association between secondary traumatic stress and post-traumatic growth—indicating that the positive relationship between secondary traumatic stress and post-traumatic growth is conditional upon low levels of burnout—organizational interventions should focus on mitigating burnout. This approach is essential not merely as a generic wellness goal but to address the psychological conditions under which professional distress and growth are linked. Practically, this involves implementing systematic caseload weighting tools that account for trauma density to ensure practitioners have sufficient cognitive downtime to prevent cognitive exhaustion that blocks the distress-to-growth transition [43]. In addition, establishing supportive policy infrastructure such as designated mental health days, accessible psychological first aid, and administrative recognition can help foster institutional trust and preemptively address burnout-induced cognitive bottlenecks.

At the supervisory and peer level, our finding that perceived social support exhibits a strong, positive direct association with both resilience and post-traumatic growth, yet does not moderate the stress–adaptation relationships, has critical practical implications. This empirical pattern suggests that while informal social support serves as an invaluable baseline stabilizer for maintaining general well-being, it is not linked to variations in how practitioners experience traumatic distress in relation to professional adaptation. Consequently, organizations cannot rely solely on peer camaraderie or general support networks to manage the complexities of workplace trauma. Instead, they should transition toward specialized, trauma-informed clinical interventions. Specifically, traditional supervision should be restructured to prioritize “reflective supervision” [44], which is specifically designed to support the structured cognitive translation of distress into professional meaning, paired with formalized, peer-led consultation groups to address isolation and reinforce collective resilience within teams [45].

At the individual level, our key finding—that compassion satisfaction negatively moderates the stress–adaptation pathways—exhibits a paradoxical “buffering-ceiling” effect whereby the positive association between secondary traumatic stress and post-traumatic growth is less pronounced under conditions of high compassion satisfaction—demands a fundamental departure from conventional “one-size-fits-all” wellness recommendations. Rather than promoting generic, uncritical compassion satisfaction for all staff, individual-level interventions must be highly tailored based on the practitioner’s psychometric profile.

For social workers exhibiting low baseline compassion satisfaction, “defensive” resource-rebuilding programs such as Compassionate Mind Training (CMT) [46] and mindfulness-based stress reduction are highly critical. In this subgroup, enhancing compassion satisfaction is essential to strengthen their early-stage defensive shield, thereby helping to decouple acute traumatic distress from severe psychological impairment. Conversely, for practitioners who already exhibit high baseline compassion satisfaction, further generic compassion satisfaction-boosting is redundant and may reinforce the “ceiling effect” that limits growth experienced in the context of traumatic stress. For these highly satisfied practitioners, interventions should pivot away from emotional buffering toward advanced cognitive flexibility and mindfulness and meaning-reconstruction training [47]. This approach equips them to process occupational trauma through structured, reflective frameworks without experiencing cognitive stagnation, while acknowledging that their psychological adaptation is characterized primarily by stable baseline resources rather than distress-associated post-traumatic growth.

### 4.7. Limitations and Future Directions

Despite the theoretical and practical contributions of this study, several boundary conditions and limitations should be contextualized to guide future research. First, the cross-sectional research design limits our ability to establish definitive temporal precedence or draw firm causal inferences regarding the relationships among secondary traumatic stress, burnout, compassion satisfaction, perceived social support, and positive adaptation outcomes (resilience and post-traumatic growth). Although our moderated relationships are robustly grounded in the Job Demands–Resources (JD-R) model and Risk–Resilience frameworks, psychological adaptation in high-stress environments can be reciprocal or bidirectional. Future studies should employ longitudinal, panel, or prospective designs to more rigorously map the developmental trajectories and directionality of these variables over time.

Second, while all constructs were assessed using psychometrically validated self-report measure instruments, the reliance on subjective questionnaires remains susceptible to potential recall bias, social desirability, and common method variance (CMV). To address these potential methodological artifacts, we implemented proactive procedural controls (e.g., ensuring complete participant anonymity and utilizing scales with distinct response formats). Furthermore, a post hoc Harman’s single-factor diagnostic indicated that CMV was highly unlikely to threaten the validity of our findings. Nonetheless, incorporating multi-source assessments (e.g., supervisor evaluation) or mixed-method designs in future research would further strengthen methodological triangulation.

Third, resilience was measured using the 2-item Connor–Davidson Resilience Scale (CD-RISC-2). Despite acceptable reliability (α = 0.76), a two-item instrument cannot fully capture the multidimensionality of resilience, risking limited content and construct validity while introducing larger measurement error. Methodologically, this constraint might have attenuated the statistical power to detect a significant direct association between secondary traumatic stress and resilience. Future research should employ more comprehensive measures, such as the CD-RISC-10 or CD-RISC-25, to minimize measurement error and better capture the complexity of resilience.

Fourth, our regression models controlled only for demographic covariates, omitting key occupational variables such as years of professional experience, weekly caseload density, and the type of employing institution. These unmeasured occupational characteristics represent potential confounding factors. For example, social workers with higher caseloads or less experience may exhibit higher vulnerability to burnout and lower capacity for positive adaptation, which could confound the observed relationships among secondary traumatic stress, burnout, and adaptation indicators. Future studies should systematically collect and control for these structural and occupational covariates to enhance internal validity and to facilitate the development of more precise, context-specific workplace interventions.

Last but not least, the empirical scope of this study was limited to active social workers in China and Singapore. Given that occupational demands, organizational resources, and sociocultural coping scripts differ across global healthcare and social service systems, these findings should be generalized to other geographic and cultural contexts with appropriate caution. To enhance the external validity of our model, future research should replicate these moderated pathways within diverse cultural cohorts and alternative professional caregiving sectors.

## 5. Conclusions

This study advances the occupational trauma literature by resolving a key conceptual debate: whether resilience and post-traumatic growth are interchangeable indicators of adaptation. Our findings empirically demonstrate that they are distinct constructs characterized by different psychological mechanisms and resource patterns. Resilience functions as a capacity for psychological stability, showing robust positive associations with resource scaffolds (compassion satisfaction and social support) rather than a direct negative relationship with secondary traumatic stress. In contrast, post-traumatic growth operates as a transformative process that is paradoxically and positively associated with secondary traumatic stress, while this relationship is significantly attenuated in the presence of occupational fatigue.

These insights challenge the dominant, purely pathological paradigm that views secondary trauma as an inevitably destructive force. Instead, they support a relational, resource-oriented model of professional quality of life. From a health ecology perspective, individual adaptation is embedded within a multi-layered system in which micro-level psychological resources (resilience, compassion satisfaction), meso-level organizational support (supervision, recognition), and macro-level professional structures are collectively linked to social workers’ occupational well-being. This ecological lens moves beyond individual-level explanations and directs attention toward the systemic conditions—such as equitable resource distribution and institutional recognition—that are closely aligned with the context of secondary trauma. Rather than focusing solely on passive distress reduction, organizational interventions may benefit from a proactive, dual-focused strategy that simultaneously addresses systemic fatigue and burnout while supporting professional efficacy and social support networks to promote sustainable adaptation.

## Figures and Tables

**Table 1:** Description of demographic variables (N = 673).

Demographic		China	Singapore
		n	n
Gender	Female	227	276
	Male	92	78
Marriage Status	Single (unmarried)	107	153
	Separated	1	3
	Divorced	6	4
	Married	199	190
	Cohabited	4	0
	Widowed	2	4
Age	21–40 years old	247	240
	41–60 years old	70	109
	60+ years old	2	5

**Table 2:** Descriptive Statistics and Normality Test Results.

Constructs	Items	Mean	SD	Kurtosis	Skewness	Overall Mean	Overall SD
Secondary Traumatic Stress	STS1	3.59	0.95	−0.40	−0.30	2.42	0.57
	STS2	2.53	0.97	0.01	0.41		
	STS3	2.88	1.07	−0.54	0.35		
	STS4	2.07	0.93	0.44	0.74		
	STS5	2.33	1.01	−0.20	0.51		
	STS6	2.3	0.95	−0.24	0.37		
	STS7	2.28	0.96	−0.11	0.48		
	STS8	2.05	0.93	0.92	0.91		
	STS9	1.93	0.9	1.28	1.06		
	STS10	2.23	0.83	0.43	0.47		
Burnout	BO1	2.32	0.87	0.68	0.63	2.41	0.63
	BO2	2.31	0.87	0.28	0.46		
	BO3	2.06	0.9	0.75	0.83		
	BO4	2.32	1.04	−0.12	0.57		
	BO5	1.98	0.88	0.76	0.85		
	BO6	2.5	0.91	0.13	0.27		
	BO7	2.8	1.06	−0.30	0.26		
	BO8	2.92	1.13	−0.56	0.18		
	BO9	2.82	1.19	−0.73	0.21		
	BO10	2.08	0.84	0.48	0.60		
Compassion Satisfaction	CS1	3.98	0.81	0.72	−0.69	3.71	0.68
	CS2	3.62	0.93	−0.25	−0.29		
	CS3	3.94	0.86	0.80	−0.76		
	CS4	3.67	0.89	0.22	−0.42		
	CS5	3.67	0.89	0.24	−0.42		
	CS6	3.61	0.85	0.54	−0.42		
	CS7	3.77	0.85	0.17	−0.41		
	CS8	3.83	0.89	0.54	−0.63		
	CS9	3.05	0.99	−0.26	0.11		
	CS10	3.94	0.89	0.55	−0.72		
Perceived Social Support	PSSS1	3.65	0.86	0.28	−0.35	3.82	0.72
	PSSS2	3.91	0.92	0.10	−0.64		
	PSSS3	3.79	0.92	0.95	−0.88		
	PSSS4	3.97	0.83	0.95	−0.78		
	PSSS5	3.77	0.98	0.48	−0.8		
	PSSS6	3.84	0.94	0.74	−0.88		
Resilience	RISC10	2.77	0.82	3.40	−1.09	2.78	0.71
	RISC11	2.79	0.76	1.24	−0.57		
PTG	PTGI1	2.13	1.39	−0.76	−0.10	2.02	1.19
	PTGI2	2.43	1.51	−0.97	−0.14		
	PTGI3	2.13	1.51	−0.95	0.07		
	PTGI4	2.13	1.54	−1.01	0.07		
	PTGI5	2.06	1.5	−0.93	0.13		
	PTGI6	1.78	1.53	−0.85	0.40		
	PTGI7	2.03	1.45	−0.96	0.13		
	PTGI8	1.32	1.55	−0.55	0.84		
	PTGI9	1.97	1.5	−0.91	0.25		
	PTGI00	2.21	1.5	−0.93	0.10		

**Table 3:** Correlations Among Study Variables.

	STS	PTG	Resilience	CS	BO	PSS
STS	1					
PTG	0.18 **	1				
Resilience	−0.18 **	0.14 **	1			
CS	−0.04	0.21 **	0.46 **	1		
BO	0.45 **	−0.10 *	−0.41 **	−0.72 **	1	
PSS	−0.17 **	0.18 **	0.38 **	0.46 **	−0.45 **	1

* *p* < 0.05; ** *p* < 0.01.

**Table 4:** Multicollinearity diagnostics.

Item	VIF Value	Tolerance
Country	1.492	0.670
Gender	1.050	0.952
Age (60+)	1.230	0.813
Age (41–60)	1.208	0.828
Marital Status (Widowed)	1.065	0.939
Marital Status (Cohabited)	1.242	0.805
Marital Status (Married)	1.169	0.856
Marital Status (Divorced)	1.074	0.931
Marital Status (Separated)	1.044	0.958
STS	2.006	0.498
S_CS	3.115	0.321
S_BO	4.222	0.237
S_PSS	1.522	0.657
S_Resilience	1.429	0.700
S_PTG	1.139	0.878
STS × CS	1.740	0.575
STS × BO	1.577	0.634
STS × PSS	1.666	0.600

S_CS, S_BO, S_PSS, and S_Resilience indicate that CS, BO, PSS, and Resilience have been centered.

**Table 5:** Hierarchical regression analysis (DV = resilience, N = 673).

Variables	Step1			Step2			Step3		
	β	t	*p*	β	t	*p*	β	t	*p*
Gender	0.05	1.15	0.25	0.04	1.10	0.27	0.04	1.23	0.22
Country	0.02	0.40	0.69	−0.11	−2.82	0.01	−0.11	−2.74	0.01
**Age (Ref** **: 21–40)**									
41–60	0.12	3.09	0.00	0.05	1.41	0.16	0.05	1.45	0.15
60+	0.07	1.67	0.10	0.05	1.25	0.21	0.05	1.35	0.18
**Marital status (Ref: Single)**									
Separated	0.01	0.23	0.82	0.06	1.90	0.06	0.06	1.92	0.06
Divorced	−0.02	−0.54	0.59	0.01	0.38	0.71	0.02	0.61	0.54
Married	−0.01	−0.21	0.84	−0.04	−1.02	0.31	−0.03	−0.96	0.34
Cohabited	−0.05	−1.22	0.22	0.01	0.17	0.86	0.02	0.49	0.63
Widowed	0.00	−0.04	0.97	0.02	0.54	0.59	0.01	0.39	0.70
S_STS				−0.08	−1.79	0.07	−0.06	−1.33	0.18
S_CS				0.32	5.80	0.00	0.30	5.24	0.00
S_BO				−0.10	−1.54	0.13	−0.12	−1.76	0.08
S_PSS				0.19	4.80	0.00	0.19	4.76	0.00
STS × CS							−0.14	−3.26	0.00
STS × BO							−0.03	−0.83	0.40
STS × PSS							0.04	0.83	0.41
F	F (9, 663) = 1.559, *p* = 0.124			F (13, 659) = 20.369, *p* = 0.000			F (16, 656) = 17.455, *p* = 0.000		
R^2^	0.02			0.29			0.30		
adjusted R^2^	0.01			0.27			0.28		
ΔR^2^	0.02			0.27			0.01		

S_STS, S_CS, S_BO, S_PSS indicate that STS, CS, BO, and PSS have been centered.

**Table 6:** Hierarchical regression analysis (DV = PTG, N = 673).

Variables	Step1					Step2					Step3		
	β	t		*p*		β	t		*p*		β	t	*p*
Gender	0.05	1.38		0.17		0.04	1.13		0.26		0.05	1.20	0.23
Country	0.03	0.64		0.52		−0.06	−1.40		0.16		−0.08	−1.70	0.09
**Age (Ref** **: 21–40)**													
41–60	−0.01	−0.34		0.73		−0.01	−0.31		0.76		−0.02	−0.43	0.67
60+	0.01	0.34		0.73		0.02	0.49		0.63		0.02	0.54	0.59
**Marital status (Ref: Single)**													
Separated	0.00	−0.07		0.94		0.01	0.26		0.79		0.01	0.36	0.72
Divorced	0.03	0.86		0.39		0.05	1.40		0.16		0.06	1.66	0.10
Married	0.07	1.62		0.11		0.04	1.00		0.32		0.04	1.03	0.30
Cohabited	0.02	0.37		0.71		0.05	1.17		0.24		0.06	1.48	0.14
Widowed	−0.01	−0.34		0.74		−0.02	−0.39		0.70		−0.02	−0.41	0.68
S_STS						0.25	4.99		0.00		0.29	5.70	0.00
S_CS						0.11	1.70		0.09		0.10	1.55	0.12
S_BO						−0.09	−1.18		0.24		−0.10	−1.29	0.20
S_PSS						0.14	3.08		0.00		0.13	2.88	0.00
STS × CS											−0.15	−3.06	0.00
STS × BO											−0.13	−2.86	0.00
STS × PSS											0.03	0.62	0.54
F	F (9, 663) = 0.649, *p* = 0.755					F (13, 659) = 5.783, *p* = 0.000					F (16, 656) = 5.611, *p* = 0.000		
R^2^	R2 = 0.009					R2 = 0.102					R2 = 0.120		
adjustedR^2^	−0.005					0.085					0.099		
ΔR^2^	0.009					0.094					0.018		

S_STS, S_CS, S_BO, S_PSS indicate that STS, CS, BO, and PSS have been centered.

**Table 7:** Comparing the Moderating Effects on Resilience and Post-Traumatic Growth.

Variables	Resilience	PTG
STS	Main effect: not significant (β = −0.08, *p* = 0.07)	Main effect: significantly positive (β = 0.25, *p* < 0.01)
CS	Main effect: significantly positive (β = 0.32, *p* < 0.01) Moderating effect: significantly negative (STS × CS: β = −0.14, *p* < 0.01)	Main effect: not significant (β = 0.11, *p* = 0.09) Moderating effect: significantly negative (STS × CS: β = −0.15, *p* < 0.01)
BO	Main effect: not significant (β = −0.10, *p* = 0.13) Moderating effect: not significant (STS × BO: β = −0.03, *p* = 0.40)	Main effect: not significant (β = −0.09, *p* = 0.20) Moderating effect: significantly negative (STS × BO: β = −0.13, *p* < 0.01)
PSS	Main effect: significantly positive (β = 0.19, *p* < 0.01) Moderating effect: not significant (STS × PSS: β = 0.04, *p* = 0.41)	Main effect: significantly positive (β = 0.14, *p* < 0.01) Moderating effect: not significant (STS × PSS: β = 0.03, *p* = 0.54)

## Data Availability

Data presented in this study are available on request from the corresponding authors due to privacy and legal reasons.

## Supplementary Materials

The following supporting information can be downloaded at: https://www.mdpi.com/article/10.3390/healthcare14142060/s1.

## Abbreviations

The following abbreviations are used in this manuscript:

STS	Secondary Traumatic Stress
BO	Burnout
CS	Compassion Satisfaction
PSS	Perceived Social Support
PTG	Post-traumatic Growth
