# Supplementary material for: Secondary Traumatic Stress and Positive Adaptation Among Social Workers: The Roles of Burnout, Compassion Satisfaction, and Perceived Social Support in a Cross-Sectional Study
Source: Healthcare (Basel). 2026 Jul 9;14(14):2060. doi: 10.3390/healthcare14142060 (PMC13410019; doi:10.3390/healthcare14142060)
Supplement: Supplementary file 1 [file healthcare-14-02060-s001.zip › healthcare-4275188-supplementary.pdf]

STROBE Statement—checklist of items that should be included in reports of observational studies

	Item No.	Recommendation	Page No.	Relevant text from manuscript
**Title and abstract**	1	(*a*) Indicate the study's design with a commonly used term in the title or the abstract	1	Cross-sectional Study
		(*b*) Provide in the abstract an informative and balanced summary of what was done and what was found	1	This study adopted a cross-sectional survey design...
**Introduction**				
Background/rationale	2	Explain the scientific background and rationale for the investigation being reported	2	See 1.1. The Occupational Burden of Secondary Traumatic Stress Among Social Workers
Objectives	3	State specific objectives, including any prespecified hypotheses	3	See 1.2. Theoretical Framework and Hypothesis Development
**Methods**				
Study design	4	Present key elements of study design early in the paper	6	See 2.1. Study Design, Setting, and Participants.
Setting	5	Describe the setting, locations, and relevant dates, including periods of recruitment, exposure, follow-up, and data collection	6	See 2.1. Study Design, Setting, and Participants.
Participants	6	(*a*) *Cohort study*—Give the eligibility criteria, and the sources and methods of selection of participants. Describe methods of follow-up<br>*Case-control study*—Give the eligibility criteria, and the sources and methods of case ascertainment and control selection. Give the rationale for the choice of cases and controls<br>*Cross-sectional study*—Give the eligibility criteria, and the sources and methods of selection of participants	5	See 2.1. Study Design, Setting, and Participants.
		(*b*) *Cohort study*—For matched studies, give matching criteria and		Not applicable.

		number of exposed and unexposed<br>*Case-control study*—For matched studies, give matching criteria and the number of controls per case		
Variables	7	Clearly define all outcomes, exposures, predictors, potential confounders, and effect modifiers. Give diagnostic criteria, if applicable	7	See 2.3. Variables and Measurements
Data sources/ measurement	8*	For each variable of interest, give sources of data and details of methods of assessment (measurement). Describe comparability of assessment methods if there is more than one group	*7*	See 2.3. Variables and Measurements
Bias	9	Describe any efforts to address potential sources of bias	7	See 2.2. Bias and Study Size
Study size	10	Explain how the study size was arrived at	7	See 2.2. Bias and Study Size.

Continued on next page

Quantitative variables	11	Explain how quantitative variables were handled in the analyses. If applicable, describe which groupings were chosen and why	9	See 2.6. Statistical methods
Statistical methods	12	(*a*) Describe all statistical methods, including those used to control for confounding	9	See 2.6. Statistical methods
		(*b*) Describe any methods used to examine subgroups and interactions		Not applicable.
		(*c*) Explain how missing data were addressed	9	See 2.6. Statistical methods
		(*d*) *Cohort study*—If applicable, explain how loss to follow-up was addressed<br>*Case-control study*—If applicable, explain how matching of cases and controls was addressed<br>*Cross-sectional study*—If applicable, describe analytical methods taking account of sampling strategy	6	2.1. Study Design, Setting, and Participants
		(*e*) Describe any sensitivity analyses		Not applicable.
**Results**				
Participants	13*	(a) Report numbers of individuals at each stage of study—eg numbers potentially eligible, examined for eligibility, confirmed eligible, included in the study, completing follow-up, and analysed	7	See 2.2. Bias and Study Size
		(b) Give reasons for non-participation at each stage		Not applicable.
		(c) Consider use of a flow diagram		Not applicable.
Descriptive data	14*	(a) Give characteristics of study participants (eg demographic, clinical, social) and information on exposures and potential confounders	10	See 3.1. Demographic Description
		(b) Indicate number of participants with missing data for each variable of interest	9	See 2.6. Statistical methods
		(c) *Cohort study*—Summarise follow-up time (eg, average and total amount)		
Outcome data	15*	*Cohort study*—Report numbers of outcome events or summary measures over		Not applicable.

		time		
		Case-control study—Report numbers in each exposure category, or summary measures of exposure		Not applicable.
		Cross-sectional study—Report numbers of outcome events or summary measures	10-17	See 3.1. Demographic Description-3.6. Comparison of the Moderating Effects of Psychological Resilience and PTG
Main results	16	(*a*) Give unadjusted estimates and, if applicable, confounder-adjusted estimates and their precision (eg, 95% confidence interval). Make clear which confounders were adjusted for and why they were included	13-15	This study used hierarchical regression. The unadjusted estimates and confounder-adjusted estimates are presented in Tables 5 and 6.
		(*b*) Report category boundaries when continuous variables were categorized		Not applicable.
		(*c*) If relevant, consider translating estimates of relative risk into absolute risk for a meaningful time period		Not applicable.

Continued on next page

Other analyses	17	Report other analyses done—eg analyses of subgroups and interactions, and sensitivity analyses		Not applicable.
**Discussion**				
Key results	18	Summarise key results with reference to study objectives		
Limitations	19	Discuss limitations of the study, taking into account sources of potential bias or imprecision. Discuss both direction and magnitude of any potential bias	21	See 4.7. Limitations and Future Directions
Interpretation	20	Give a cautious overall interpretation of results considering objectives, limitations, multiplicity of analyses, results from similar studies, and other relevant evidence	17-19	See 4.1. -4.3.
Generalisability	21	Discuss the generalisability (external validity) of the study results	21	See 4.7. Limitations and Future Directions
**Other information**				
Funding	22	Give the source of funding and the role of the funders for the present study and, if applicable, for the original study on which the present article is based	22	Funding

*Give information separately for cases and controls in case-control studies and, if applicable, for exposed and unexposed groups in cohort and cross-sectional studies.

Note: An Explanation and Elaboration article discusses each checklist item and gives methodological background and published examples of transparent reporting. The STROBE checklist is best used in conjunction with this article (freely available on the Web sites of PLoS Medicine at http://www.plosmedicine.org/, Annals of Internal Medicine at http://www.annals.org/, and Epidemiology at http://www.epidem.com/). Information on the STROBE Initiative is available at www.strobe-statement.org.
